# Utilization of Malaysia EAF slags for effective application in direct aqueous sequestration of carbon dioxide under ambient temperature

**DOI:** 10.1016/j.heliyon.2019.e02602

**Published:** 2019-10-10

**Authors:** Sunday O. Omale, Thomas S.Y. Choong, Luqman C. Abdullah, Shamsul I. Siajam, Mun W. Yip

**Affiliations:** a Sustainable Process Engineering Research Center (SPERC), Department of Chemical and Environmental Engineering, Universiti Putra Malaysia, UPM, Serdang, Selangor, 43400, Malaysia; b INTROP, Universiti Putra Malaysia, UPM, Serdang, Selangor, 43400, Malaysia; c Dept. of Mechanical Engineering, Tunku Abdul Rahman University College, Malaysia

**Keywords:** Engineering, Materials science, Chemistry, Environmental science, Carbon dioxide (CO_2_), Sequestration, Environment, Electric arc furnace (EAF) slag, Mineral carbonation

## Abstract

Iron and steel industries are among the contributors of CO_2_ emission in large volume into the atmosphere, causing detrimental effects to the environment and the ecosystem at large scale. These industries also generate solid wastes in the form of electric arc furnace (EAF) slag during operations which result in about 10–15% slag wastes per ton of steel produced. In this study, the EAF slags from an iron and steel-making factory in Klang, Malaysia was utilized for CO_2_ sequestration through direct aqueous mineral carbonation. According to the surface area analysis, the fresh EAF slag has a mesoporous structure, its elemental composition shows the presence of 20.91 wt.% of CaO that was used for the sequestration of CO_2_ through carbonation. The sequestration capacity was found to be 58.36 g CO_2_/kg of slag at ambient temperature in 3 h, with the liquid/solid (L/S) ratio of 5:1 and using <63μm particle size. Moreover, the shrinking core model (SCM) was used to analyze the solid-fluid reaction in a heterogeneous phase and the CO_2_ sequestration shows to be controlled by the product layer phase. The EAF slag is demonstrated to have the potential of CO_2_ sequestration at ambient temperature.

## 1 Introduction

Iron and Steel industries are among the major contributors of gaseous carbon dioxide (CO_2_) emission in large volume into the atmosphere, thereby causing serious environmental challenges (Tian et al., 2018). The mitigation of this major greenhouse gas have become a necessity, since the menace is due to man's activities in the area of development through industrialization (Styring et al., 2011). With the advent and challenges that come along with modern civilization, the emitted CO_2_ had an unprecedented increase from pre-industrial period of 277 ppm to more than 400 ppm presently (Ukwattage et al., 2017). This make the natural sequestration (weathering) unable to cope, causing a dreadful damage to the environment. It is therefore imminent, to come up with means of mitigating the release of CO_2_ by these industries.

Apart from emitting large amount of CO_2_, some industries such as steel industries also produces solid wastes in the form of slag during operations (Huijgen et al., 2005). The slag can be utilized to sequestrate the CO_2_ into a permanent state as mineral carbonate (Ukwattage et al., 2017). There are several operations being used in the production of iron and steel, such as blast furnace, basic oxygen furnace, electric arc furnace (EAF) and ladle furnace. These processes uses flux agents like, lime (CaO) or Mg/Ca (CO_3_)_2_ to reduce impurities, such as (Al_2_O_3_, SiO_2_, MnO, P_2_O_5_, C_r2_O_3_ and CO_2_) during steelmaking (Yildirim and Prezzi, 2011), as a result slag is generated as by-products. The slag from EAF production is between 10-15% per ton of steel (Zhang et al., 2016; Bankole et al., 2011) and has basically between 25 – 47 wt.% composition of CaO (Revathy et al., 2016; Hosseini et al., 2016; Doucet, 2010). Several studies have shown that the presence of calcium oxide (CaO) in the steel slag, is a key in mineral carbonation through carbon sequestration (Ukwattage et al., 2017; Zhang et al., 2016; Revathy et al., 2016; El-Naas et al., 2015; Chang et al., 2011) also using it in developing CaO based CO_2_ sorbent through calcium looping process for gaseous CO_2_ capture (Tian et al., 2016a, Tian et al., 2016b) (Tian et al., 2015). In previous studies, sequestration capacity of 82 g CO_2_/kg of slag with liquid to solid ratio (L/S) 10:1, 6 bar, 3 h was reported by Revathy et al. (2016) with CaO in fresh slag chemical composition of 28.27 wt %. Meanwhile, Li et al. (2012) had a sequestration report of 77 g CO_2_/kg of steel slag under ambient temperature in 2 h, with a gas pressure of 15 bar and liquid-solid ratio of 50 L/kg having a CaO in fresh slag of 43.34 wt%. Ghacham et al. (2016) had sequestration capacity of 46 g CO_2_/kg of slag and 52 g CO_2_/kg of slag at L/S ratio of 4:1 and 10:1 under ambient temperature. It was achieved with 10.68 bar in 10 min reaction time and CaO wt % of 33.19 in fresh slag chemical composition. Baciocchi et al. (2015) had a sequestration capacity of 280 g CO_2_/kg of EAF steel slags in 24 h, at 100 °C, with CO_2_ pressure of 10 bar, using L/S ratio of 5:1 and CaO of 35.2 wt %. Huijgen et al. (2005) was able to sequestrate 185 g CO_2_/kg of slag after 30 min at a pressure of 19 bar, 100 °C with particle sizes of <38 μm steel slag.

Therefore, in solid-fluid reaction of heterogeneous material, to establish the process of direct mineral carbonation using slag, the need to evaluate the extent of its kinetics is important. Usually, the shrinking core model (SCM) is used to analyse the solid-fluid reaction in a heterogeneous phase. The basic notion in shrinking core model is that, reactant diffused through the fluid film to react with the outer part of the (solid) slag, the reactant (chemical) reacting with the surface of the solid, then move gradually back into the main body of the fluid with the ash layer product, thereby having an unreacted core in the solid and making its size to shrink in the course of carbonation process (Jo et al., 2017; Tu et al., 2015; Ajemba and Onukwuli, 2012).

In the present study, accelerated mineral carbonation using EAF slag with 20.91 wt.% of CaO composition from a steel factory located in Klang, Malaysia to sequestrate CO_2_ was explored. Also, the permanency of CO_2_ storage at ambient temperature with optimized operating conditions such as pressure, liquid-to-solid ratio (L/S) and reaction time were investigated. The surface area, functional groups, surface morphology, elemental composition, mineral phase composition and thermal decomposition investigations were also performed. Finally, the extent of its carbonation kinetics was studied, using the shrinking core model (SCM).

## 2 Materials and methods

### 2.1 Preparation of the EAF slag sample

The EAF slag obtained from the iron and steelmaking factory Klang, Malaysia was about 20 mm in size. The sample was crushed with the laboratory roll crusher and further reduced by using ball mill. The sample was sieved with mesh of different aperture to various sizes, the particle sizes of <63μm was used. The steel slag was washed with distilled water to remove any debris and dried at 105 °C for the period of 24 h in an oven. The CO_2_ used was 99.99% pure gas of analytical grade.

### 2.2 Carbonation procedure

**Fig. 1:**
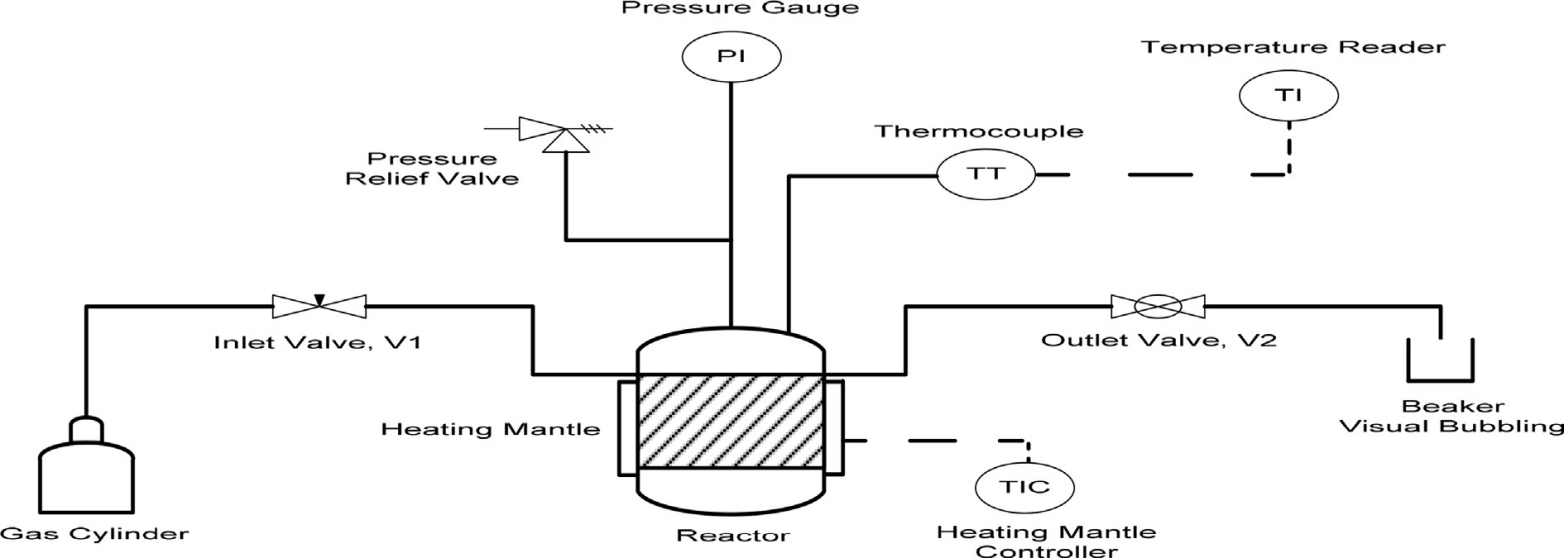
Schematic diagram of the setup.

A batch reactor made of stainless steel of 1.4 L capacity that is equipped with a gas inlet and pressure indicator was used for the CO_2_ sequestration. Fig. 1 shows the schematic diagram of the reactor setup. The experiment was conducted at various liquid (ml)/solid (gram), L/S ratio of 2:1, 5:1 and 10:1. Initially, the top cap of the reactor was opened, and the slag introduced with the required volume of distilled water. The cap was closed with Polytetrafluoroethylene (PTFE) seal, the reactor flange surface was cleaned before closing the cap and gradually screwed to tighten evenly. The reactor was purged to remove air from within by opening inlet and outlet valves and introducing the CO_2_ gas into the system for few minutes. Both valves were closed, gradually the gas inlet was open until the required pressure (5 bar) was reached, then it was closed. The system was operated at ambient temperature and placed on a magnetic stirrer to stir at 500 rpm. At the end of each of the reaction time 0.5, 1, 2, 3 and 4 h, the reactor was depressurized and disassembled. After the experiment, the slurry was decanted and filtered through Whatmann filter paper (Filtres Fioroni pore size 5 μm code 113) and allowed to dry at a temperature of 105 °C in an oven until the water is completely dried. The experiments were conducted in triplicate throughout and an average value was used.

The slag specific surface area was measured with automated surface area and porosity analyzer (Micromeritics ASAP, 2020) by the adsorption and desorption of N_2_ at 77 K. Before analysis, the sample was degassed at 250 °C for 4 h. The surface area and pore size distribution were obtained using the BET and Barrett-Joyner-Halenda (BJH) calculations. The FTIR analysis was performed on the sample to determine the surface functional groups in range 4000–400 cm^−1^. A Perkin Elmer Spectrum (100 FT-IR Spectrometer) with PIKE MIRACLE ATR (attenuated total reflection) attachment was used to record the spectra. The morphology and elemental composition of the slag was observed and analyze with SEM/EDX (Hitachi Co., Japan, Model No. S3400N). The X-ray diffraction (XRD) for mineral composition analysis was performed with an X'Pert Philips PW3040 diffractometer using Cu Kα radiation (2θ range = 10^°^–90 ^°^; step = 0.05 ^°^ 2θ; time per step = 0.2 s). The XRD patterns were indexed according to the Powder Data File database (PDF, 2000, International Centre of Diffraction Data, Pennsylvania). The average sizes of the crystallites were determined using the Scherrer formula, D = 0.9λ/βcos θ, where λ is the wavelength of the Cu Kα radiation, β is the full width at half maximum (in radians), 0.9 is the shape factor for spherical particles and θ is the angle of the diffraction peaks. The thermal analysis was performed using a thermo-gravimetric (TGA) analyzer (Mettler Toledo equipment), at a rate of 10 °C/min to 1000 °C under a N_2_ flow of 25 ml/min.

Eq. (1) was used to calculate the carbon (CO_2_) uptake.

(1) $$CO_{2}\left(wt\%\right)=\;\frac{△m_{500{}^{\circ}C-900{}^{\circ}C}}{m_{105{}^{\circ}C}}\times \;100$$

Where, CO_2_ (wt%) is the carbon uptake, m_105_°_C_ represents the carbonate slag weight loss when heated to 105 °C and Δm_500°C–1000°C_ represent weight loss when the calcium carbonate was heated from 500 °C to 1000 °C, (Zhang et al., 2016; El-Naas et al., 2015; Huijgen et al., 2005). The CaCO_3_ decomposition is as shown in Eq. (3) and using the values from the TGA decomposition graph at different temperatures, the weight loss values were calculated.

The carbon uptake in gram of CO_2_ per kg of slag is calculated when a kg of slag is used. While the sequestration efficiency (SE) of CO_2_ was calculated using Eq. (2) with the maximum sequestration of CO_2_ wt% uptake from Eq. (1) and the maximum CO_2_ theoretical sequestration capacity (%CO_2_) from Eq. (4).

(2) $$CO_{2}Sequestration\;efficiency\left(\%\right)=\frac{\left(CO_{2}wt\%\right)}{\%CO_{2}}\times \;100\;$$

## 3 Results and discussion

### 3.1 Characterization results

**Fig. 2:**
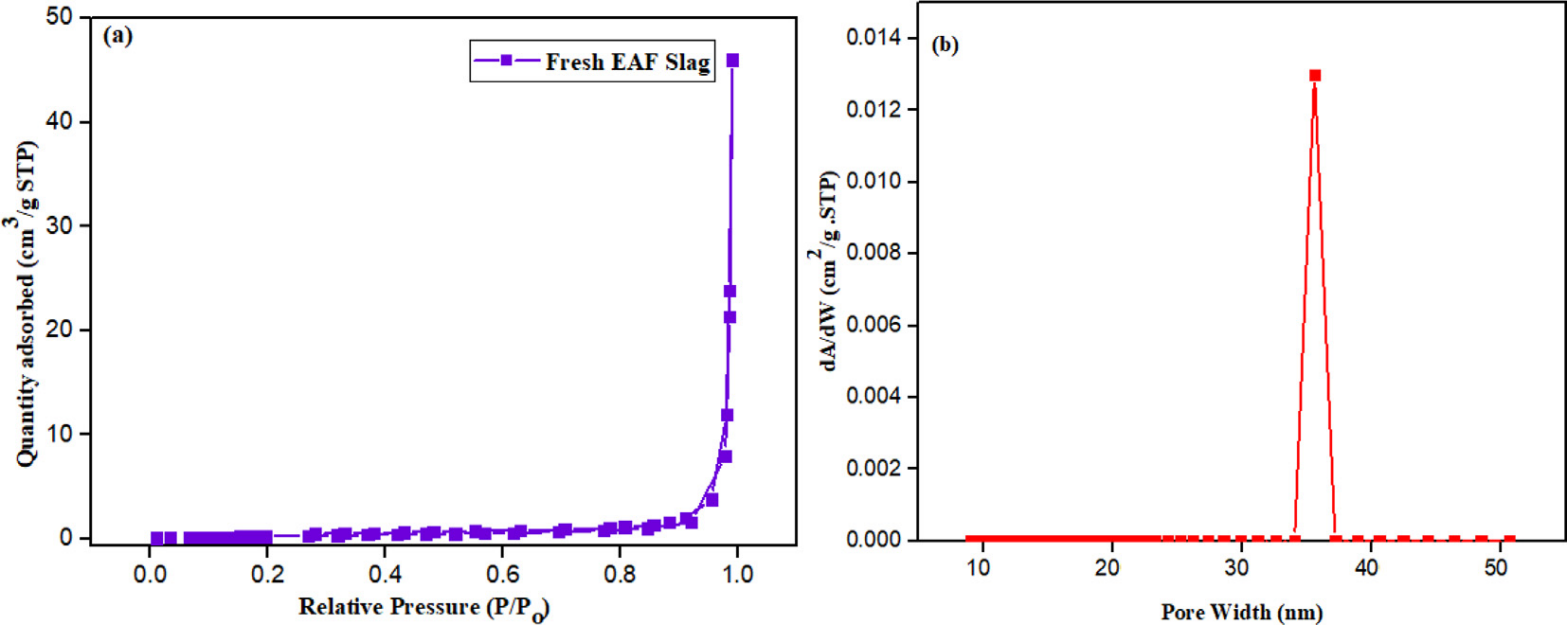
(a) N2 adsorption–desorption plots of fresh EAF slag and (b) pore size distribution plots.

The BET specific surface area (S_BET_) analysis of the EAF slag before carbonation was performed using nitrogen gas analysis adsorptive. The result shows S_BET_ of the slag to be 1.2 m^2^/g, pore volume 0.99 cm³/g and pore size 203 nm Fig. 2 shows the N_2_ adsorption–desorption and pore size distribution plots of the fresh EAF slag. Obviously, the specific surface area, total volume and pore size distribution of an adsorbent are its important characteristics (Rađenović et al., 2013).

Accordingly, Fig. 2(a) shows the N_2_ adsorption–desorption plot, it indicate a type IV isotherm, which illustrates the qualities of the porous media to have mesoporous structure, based on the International Union of Pure and Applied Chemistry classification. This result was supported by Fig. 2(b) which shows pore size distribution based on the BJH calculation as 37 nm, which implies the mesoporous nature of the fresh slag, this finding is similar to the work of Irena et al. (2019).

**Fig. 3:**
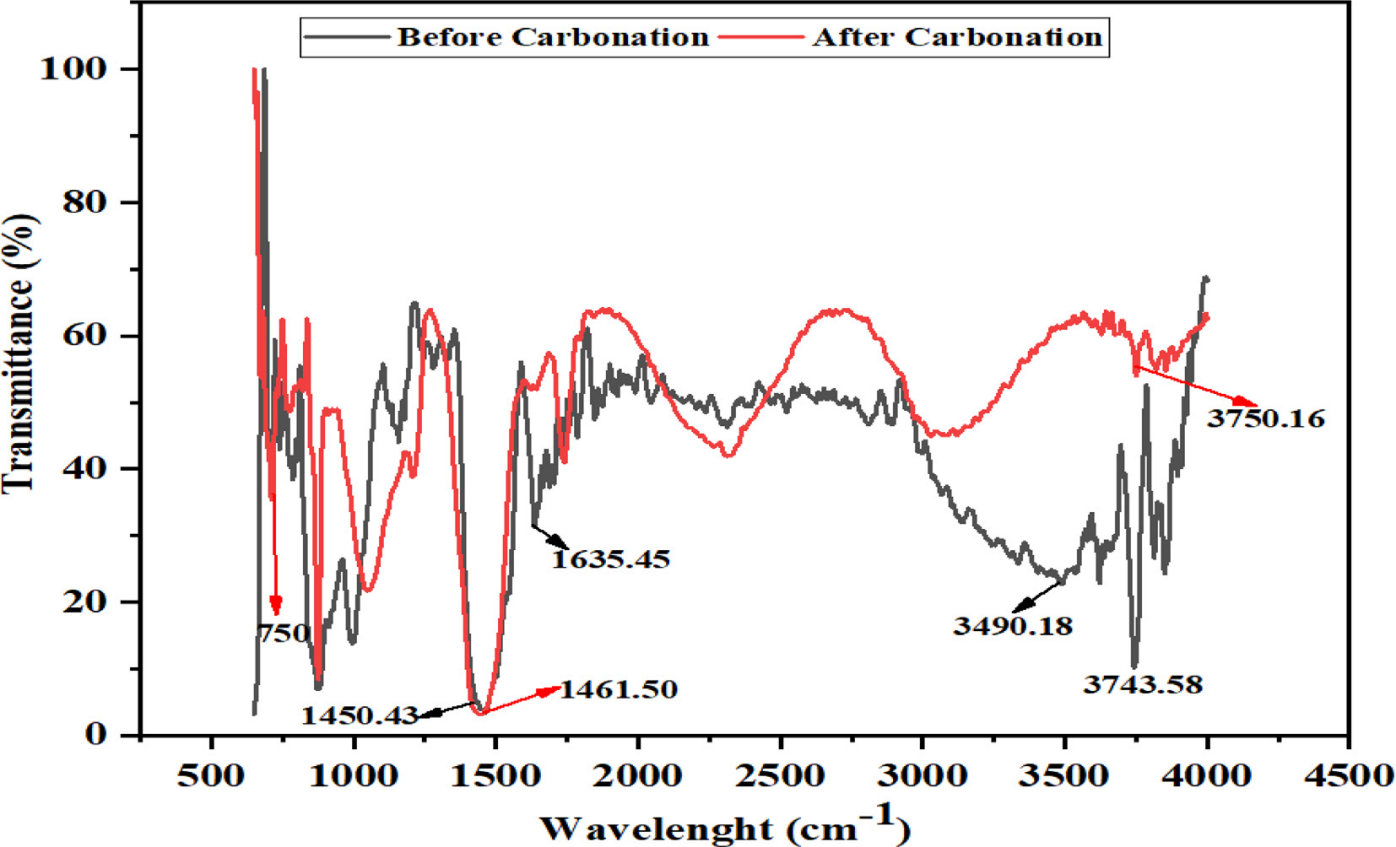
The FTIR spectra before and after carbonation.

The FTIR spectrum is used, to give information about the presence or non-existence of specific functional groups in a given sample. From Fig. 3 of the analyzed slag, shows the following broad bands were displayed: 875-1480 cm^−1^ corresponding to C–O group and 2000-3000 cm^−1^ band showing the existence of carbonate group and aromatic of double bond C=C with medium-weak intensity multiple bands. The strong intensity vibration confirmed the initial carbonation of fresh slag due to natural weathering and after carbonation, there was a slight increase of the sample peak to 1462 cm^−1^. The medium-weak intensity of 2000–3000 cm^−1^ also shows the present of MgO (Rađenović et al., 2013), (Revathy et al., 2016) The broad band of 3500–3750 cm^−1^ stretch free with strong and sharp intensity shows the presence of O–H group. The vibration band between 900–1200 cm^−1^ shows also to have Si–O group, its stretch confirmed silicate present with little volume of water in both dry fresh slag and its carbonated sample (Revathy et al., 2016). In considering the adsorption nature of the slag, adsorption is concern with the happening on the surface of a material, making the adsorbate molecules attracted and held by the adsorbent on its surface till equilibrium is established among the molecules. The interaction between the adsorbent and the adsorbed molecules is key in the adsorption behavior of a material. However, to understand the adsorption mechanism of the slag, the surface area of the adsorbent is crucial. Also, the electrostatic adsorbent and adsorbate interaction strongly depend on the functional group existence at the surface, and the narrowest micro-porosity nature in an improved adsorption potential is likely to be good enough to retain and adsorb ions (Rađenović et al., 2013). There are many minor functional groups whose intensities are very low and therefore would not have much influence during carbonation. While in the spectra after carbonation, most of the functional groups would have disappeared/consumed or minimized as a result of the formation of a new product. This is similar to the finding of Revathy et al. (2016).

**Fig. 4:**
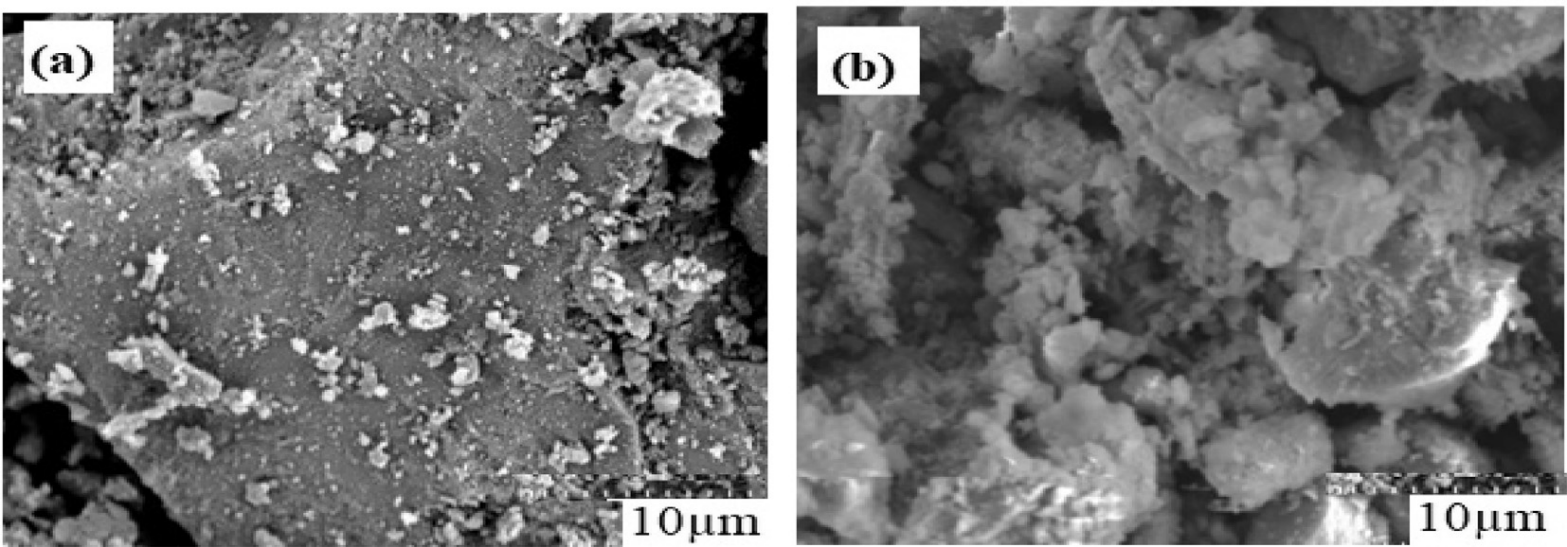
SEM images of Klang EAF slag (a) Before carbonation (b) After carbonation.

The SEM image in Fig. 4 (a) shows the agglomerated particles having irregular shapes and sizes with rough surfaces. The amount of the attached components become most pronounced making the surface of the material to be irregular and rough because of the minor groups. Hosseini et al. (2016) found that the irregularity was due to the presence of minor groups that has disappeared/consumed or minimized after carbonation.

Furthermore, Fig. 4 (b) shows the carbonation of the slag after the reaction time with the particles giving a new image of the carbonate having cubic structures, most likely because of the formation of calcite particles in the carbonation. The aragonite was also noticed because of the presence of little magnesium in the carbonation process. This confirmed the XRD analysis results obtained. This can be seen from the formation and changes in the surface morphology, showing agglomerated, rough and coarse, surfaces (Ukwattage et al., 2017; Revathy et al., 2016; Zhang et al., 2016). The SEM image result (b) implied the possibility of using the slag for direct carbonation purpose.

**Fig. 5:**
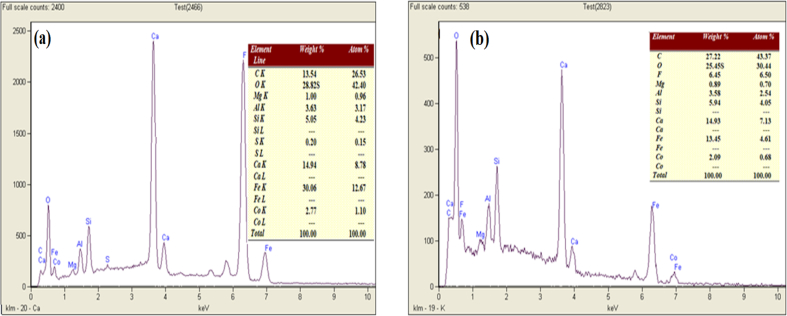
EDX peaks of the EAF slag (a) Before carbonation (b) After carbonation.

The EDX result of the slag sample shows the composition of fresh and carbonated material as presented in Fig. 5 (a) and (b) respectively. The fresh slag elemental composition was 20.91% of CaO. After carbonation, the peak of the carbon increases as compared to the fresh sample. The EDX peak increase results further confirms the usability of the present material for carbonation.

**Fig. 6:**
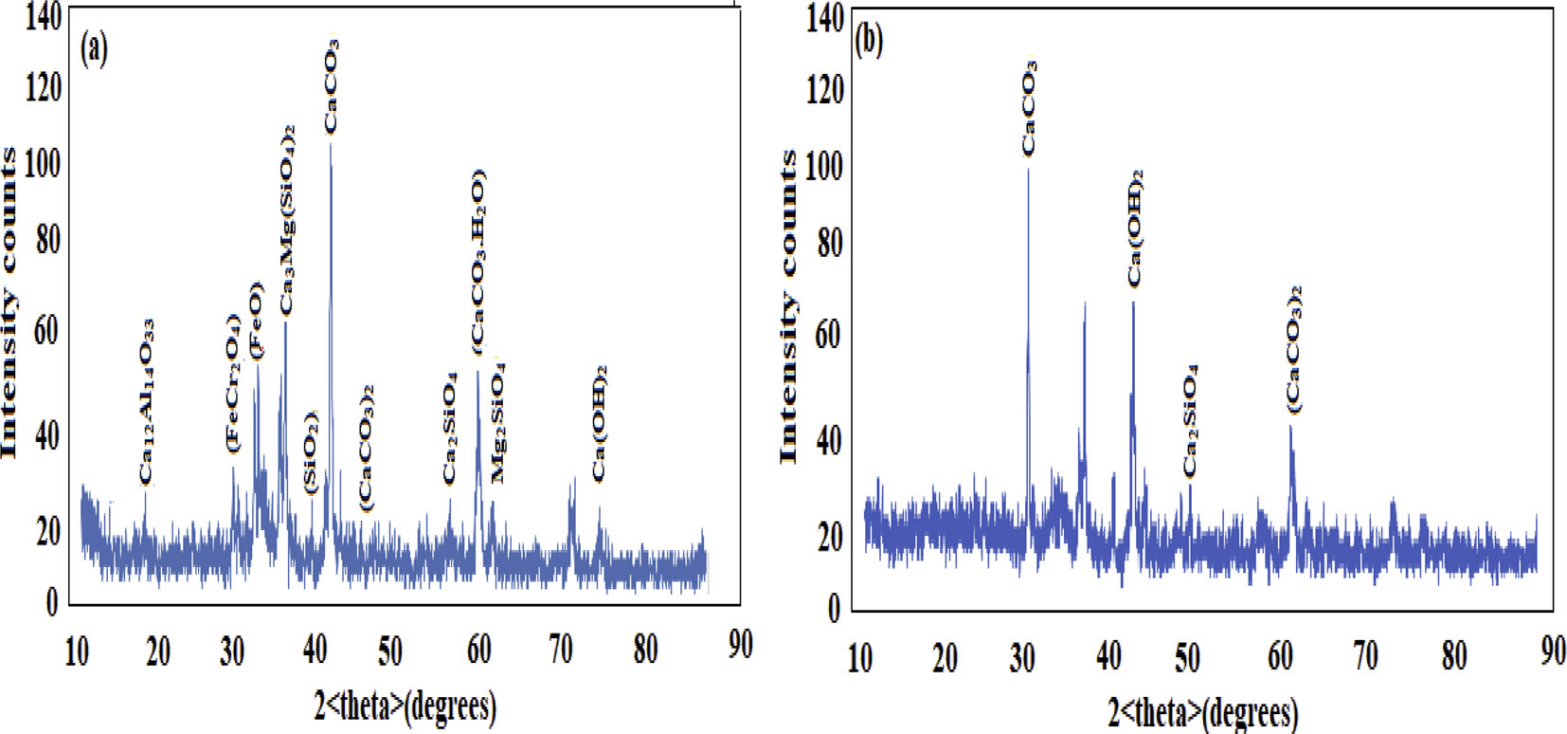
(a) XRD pattern before carbonation (b) after carbonation.

The XRD patterns of the slags before and after carbonation are presented in Fig. 6 (a) and (b). Before carbonation in Fig. 6(a), the results obtained shows the presence of Mayenite (Ca_12_Al_14_O_33_), Forsterite (Mg_2_SiO_4_), Calcite (CaCO_3_), Larnite (Ca_2_SiO_4_), Merwinite {Ca_3_Mg(SiO_4_)_2_}, Magnesite (MgCO_3_), Hematite (Fe_2_O_4_), Monohydrocalcite (CaCO_3_.H_2_O), Portlandite {Ca(OH)_2_}, Wustite (FeO), Chromite (FeCr_2_O_4_), Quartz (SiO_2_) and Angenite (CaCO_3_)_2_. This results agrees with the earlier findings (Revathy et al., 2016; Bankole et al., 2011; Venkateswaran et al., 2016).

The calcium phase contained majorly calcite, larnite, portlandite, merwnite and mayenite. After carbonation in Fig. 6 (b), the phase composition formed differs from the un-carbonized sample indicating that the calcite has a predominant phase thus showing a major peak while other minor phases include aragonite (CaCO_3_) and dolomite (CaMg(CO_3_)_2_.

Peaks intensity of mayenite, larnite and merwinite were reduced, while others were missing or consumed in the carbonized phase. These minerals all donated to the calcite formation in aqueous carbonation, chiefly among the most reactive are merwinite and larnite implying that the slag from iron and steel can be used to reduce CO_2_ through mineral carbonation (Tu et al., 2015).

**Fig. 7:**
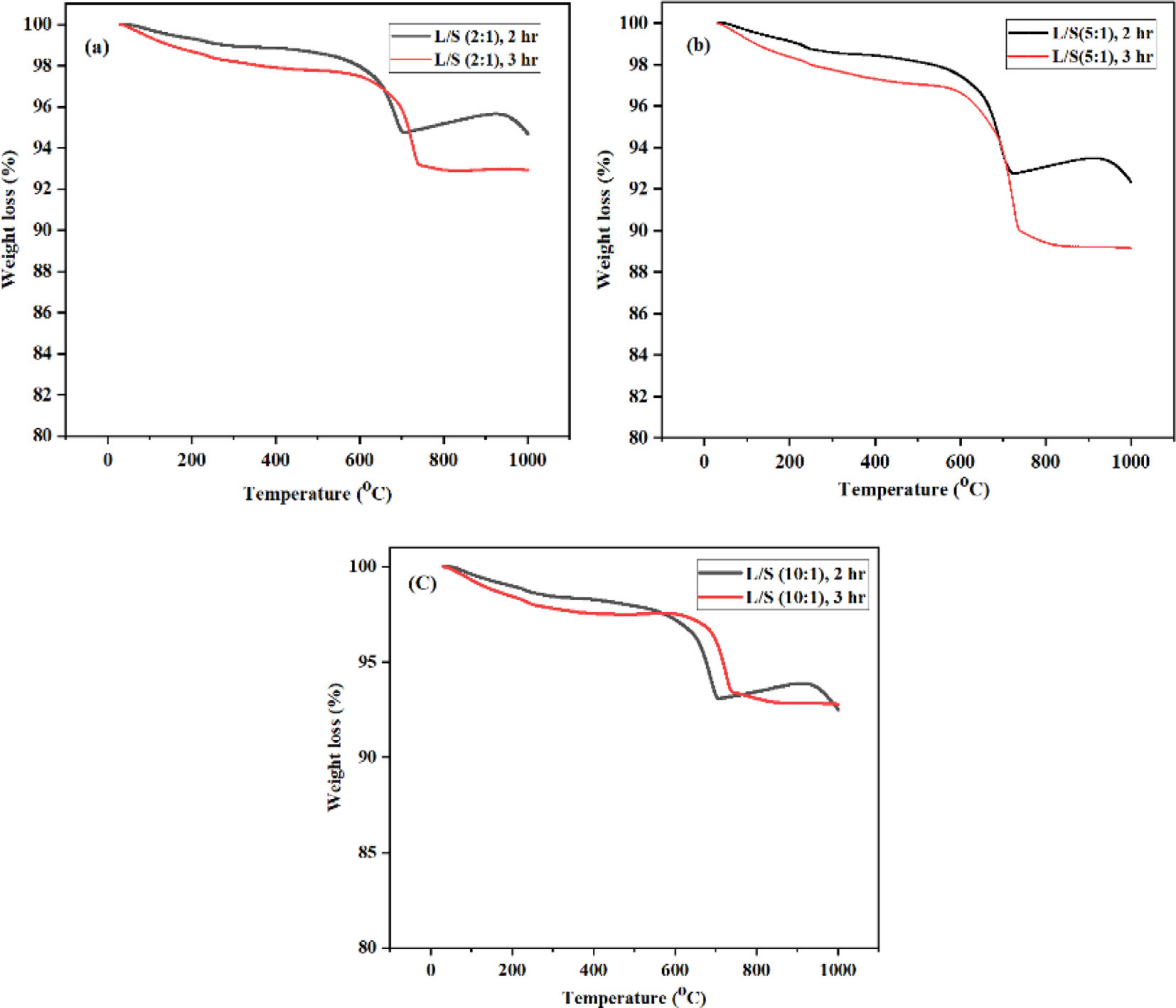
The thermo-gravimetric analysis plots at (a) L/S (2:1), (b) L/S (5:1) and (c) L/S (10:1).

The weight loss of the slag was obtained from the curve analysis of the thermo-gravimetric, with the range from 25 – 1000 °C. The carbonated material decomposed at 700 °C as shown in Fig. 7 (a,b and c) for various L/S ratio.

The carbonated slag weight losses was noticed on the TGA analysis curve which include three parts mainly; the moisture evaporation at 25–100 °C, organic carbon loss and magnesium carbonate decompositions at 105–500 °C while at 500–1000 °C, the decomposition of the carbonate of calcium was effected from the reaction of calcium carbonate as shown in Eq. (3):

(3) $$CaCO_{3}\to CaO+CO_{2}$$

The efficiency of carbon sequestration is calculated using the Stenoir's stoichiometric in Eq. (4) for the total theoretical carbon uptake %CO_2_. It is the content of fresh sample that depend on the metal oxides composition present in the fresh slag (Revathy et al., 2016; El-Naas et al., 2015).

(4) $$\%CO_{2}=0.785\left(\%CaO-0.56\%CaCO_{3}-0.7\%SO_{3}\right)+1.091\%MgO+0.71\%Na_{2}O+0.468\%K_{2}O$$

The values from Table 1 were used in the simplified Stenoir stoichiometric Eq. (5) for the calculation of the total theoretical value (%CO_2_):

(5) $$\%CO_{2}=0.785\left(\%CaO-0.7\%SO_{3}\right)+1.091\%MgO$$

**Table 1:** EDX elemental composition analysis of fresh slag before carbonation.

Formula	C	MgO	Al_2_O_3_	SiO_2_	SO_3_	CaO	Fe_2_O_3_	CO	Total %
Composition	13.54	1.65	6.86	10.79	0.50	20.91	42.98	2.77	100

Eq. (5) was used to calculate the maximum theoretical sequestrated (%CO_2_) capacity which was found to be 179.4g CO_2_/kg of slag, by assuming that the whole quantity of Ca/Mg would have been leached from the wastes (slag) and carbonated. The amount of sequestration is the basic characteristic of the waste (slag), The sequestration efficiency (SE) of CO_2_ was calculated using Eq. (2) with the maximum sequestration of CO_2_ wt% uptake from Eq. (1) and the maximum CO_2_ theoretical sequestration capacity (%CO_2_) in Eq. (5).

### 3.2 The effects of L/S ratio and time

**Fig. 8:**
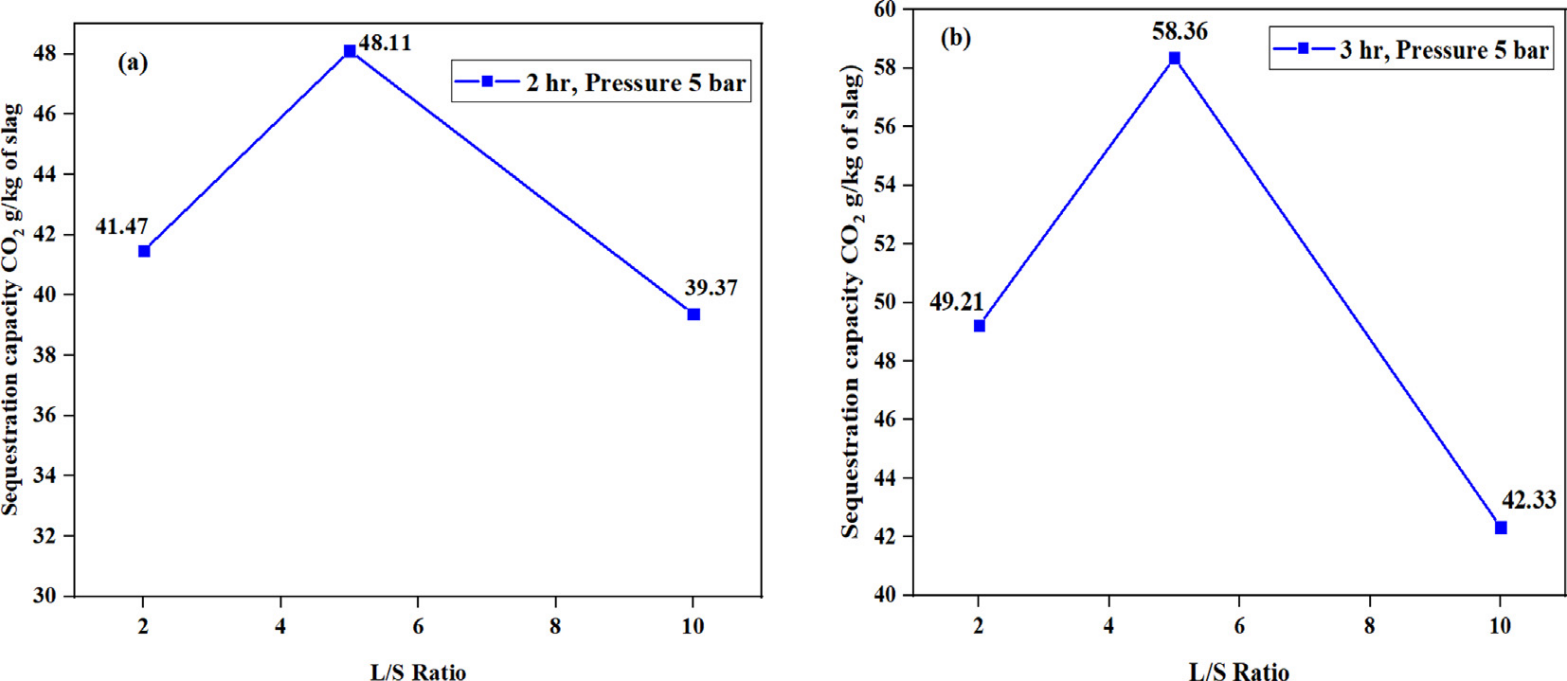
Sequestration capacity as a function of L/S ratio after **(a)** 2 h and **(b)** 3 h at ambient temperature with the pressure of 5 bar.

Fig. 8, shows the extent of CO_2_ sequestration with respect to the L/S ratio and reaction time. The experiments were carried out under fixed pressure of 5 bar and at ambient temperature. It can be seen that sequestration of CO_2_ increases as the L/S ratio increases, from 2:1 to 5:1 but reduces as the L/S ratio increases to 10:1. From Fig. 8 a, the 2 h CO_2_ sequestration capacity for L/S ratio 2:1 was 41.47 ± 0.85 while for 5:1 was 48.11 ± 4.81 and 10:1 was 39.37 ± 0.24 g/kg of slag, respectively. In Fig. 8 b, as the time proceeded to 3 h, the sequestration capacity was observed to be 49.21 ± 1.00, 58.36 ± 5.84 and 42.33 ± 0.86 for the ratio 2:1, 5:1 and 10:1, respectively. This shows that high L/S ratio like 10:1 has low values due to much water that has prevented the movement of CO_2_ gas molecules (diffusion) to contact the Ca ions for the carbonation. The sequestration for L/S ratio 5:1 was the highest out of the three different ratio studied under the same conditions, as shown in Fig. 8 b.

The extent of carbonation within a system depend largely on the amount of calcium oxide present in the slag tested and the volume of water for leaching action on the calcium molecules from the slag in the slurry within the reactor based on Eqs. (6) and (7) (Ukwattage et al., 2017; Revathy et al., 2016; Tu et al., 2015).

(6) $$CO_{2}+H_{2}O\to H_{2}CO_{3}\to H^{+}+HCO_{3}^{-}$$

(7) $$Ca^{2+}+HCO_{3}^{-}\to CaCO_{3}+H^{+}$$

**Fig. 9:**
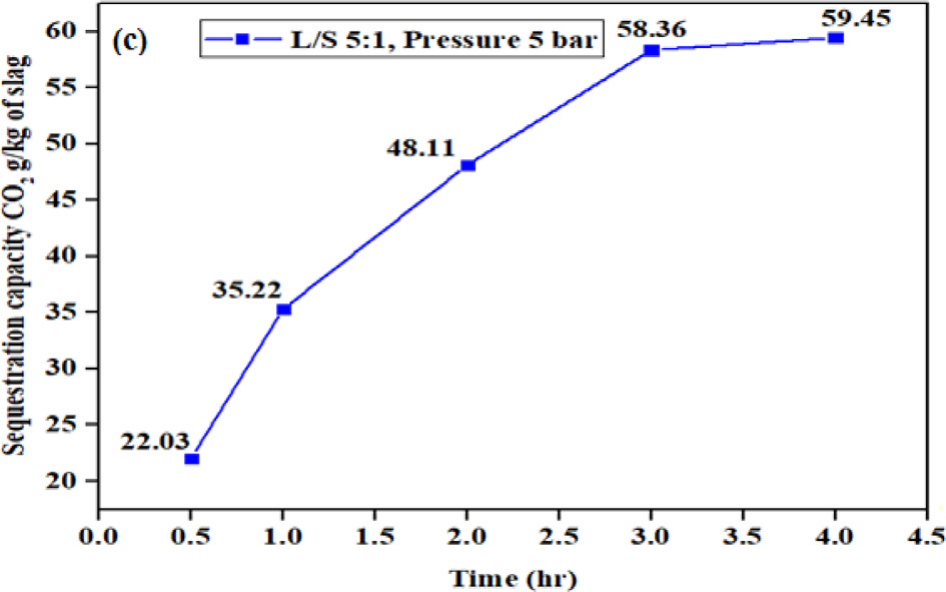
Sequestration capacity of EAF slag against reaction time.

According to these equations, aqueous carbonation in a direct phase involves CO_2_ reacting with water to produce carbonic acid (H_2_CO_3_) which will liberates the alkaline metal ion and then react with bicarbonate to form carbonate. Fig. 9 shows the plot of sequestration capacity of EAF slag against the reaction time.

The sequestration capacity increases with reaction time as observed in Fig. 9. However, the increase in sequestration capacity for reaction time of 3 h–4 h was insignificant, that is, from 58.36 ± 5.84 g to 59.45 ± 5.95 g CO_2_/kg of slag. This shows that the carbonation has reached its peak and any further sequestration will be very slow due to the formation of carbonated layer on the core surface of the slag which has prevented the gas movement and further leaching of Ca ions from the slag, causing passivation (Revathy et al., 2016; Tu et al., 2015; Chang et al., 2011).

### 3.3 The effect of pressure on carbonation

**Fig. 10:**
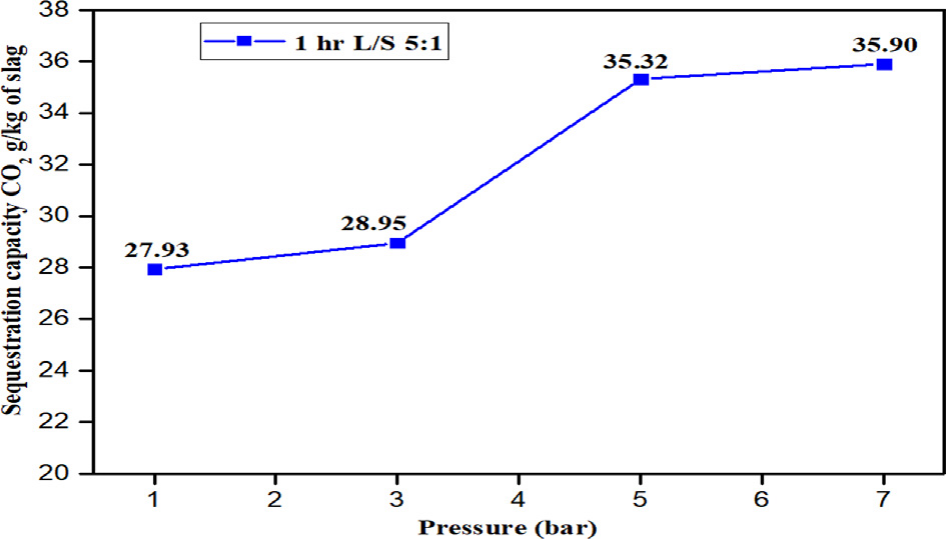
Effects of pressure on carbon sequestration under the reaction time of 1hr, at ambient temperature and L/S ratio 5:1.

The effect of pressure variation on carbon sequestration is shown in Fig. 10 for the pressures of 1, 3, 5 and 7 bar. The effect of CO_2_ initial pressure on mineral carbonation was studied for the L/S ratio of 5:1 at a reaction time of 1 h under ambient temperature. High pressure means more concentration of gas molecules will be available for dissolving in water for carbonation. Therefore, as the pressure increases to 5 bar, the carbon sequestration capacity by the slag also increases to its peak as presented in Fig. 10. More so, as the pressure increases the carbon uptake also increases to a point with sequestration capacity of 35.32 g CO_2_/kg of slag.

Any further pressure increase in the reactor after 5 bar, shows no appreciable increase in the sequestration capacity because of the CO_2_ molecules have reached an equilibrium state in the reactor as a result of the gas saturation in the water and the unoccupied space (Ghacham et al., 2016; Tamilselvi Dananjayan et al., 2016). This indicates that increase in CO_2_ pressure in the carbonation process do not mean higher CO_2_ sequestration after a point is reached as shown in Fig. 10 and as observed in the previous findings (Ukwattage et al., 2017; Revathy et al., 2016).

The sequestration capacity as reported earlier (Baciocchi et al., 2015; Huijgen et al., 2005) were higher as compared to this study. However, the chemical composition, high temperature, pressure and reaction time may be the shortfalls of the previous findings prospect for industrial application, due to the high energy consumption requirement. In the present study, at ambient temperature with a pressure of 5 bar, L/S ratio of 5:1 and in 3 h, the sequestration capacity obtained was 58.36g CO_2_/kg of slag having CaO 20.91 % and MgO 1.65 % weight in fresh elemental composition respectively. The result shows moderate conditions in direct aqueous CO_2_ sequestration.

### 3.4 Kinetics model of the carbon sequestration

The use of shrinking core model (SCM) in the reaction process of aqueous mineral carbonation will lead to better understanding of the behaviour in a heterogeneous reaction of liquid – solid which could take either of this rate controlling step (i) the diffusion of the reactant ions (carbonic acid) $CO^{2-}and\;HCO_{3}^{-}$ through the fluid film to the surface of the slag solid particles, (ii) the reaction of the reactant with the surface of the solid by diffusing and penetrating through the pores to the unreacted core and (iii) the movement of the product layer (CaCO_3_), $Ca^{2+}$ion with the carbonic acid ions at the solid particles surface back into the main body of the fluid (Octave Levenspiel, 1999). Also, with respect to the SCM, there were kinetic equations used for different control rate phases in the study:

(i) Fluid film diffusion controlled process

(8) $$k_{1}t=x$$

(ii) Controlled process of its chemical reaction

(9) $$k_{2}t=1-{\left(1-x\right)}^{1/3}$$

(iii) The product layer controlled process through diffusion

(10) $$k_{3}t=1+2\left(1-x\right)-3{\left(1-x\right)}^{2/3}$$

Where, $k_{1}t,\;k_{2}t\;and\;k_{3}t$ is the rate constant, t is reaction time (min), x is the carbon sequestration capacity of the EAF slag (Octave Levenspiel, 1999).

The kinetics conditions for the experiment were fixed as follows, mass ratio of liquid-solid (L/S) of 5:1, stirring speed of 500 r/min and particles (slag) size <63 μm, respectively. The carbon sequestration of the EAF slag in the experiment was conducted under ambient temperature at different timing as shown in Fig. 9.

**Fig. 11:**
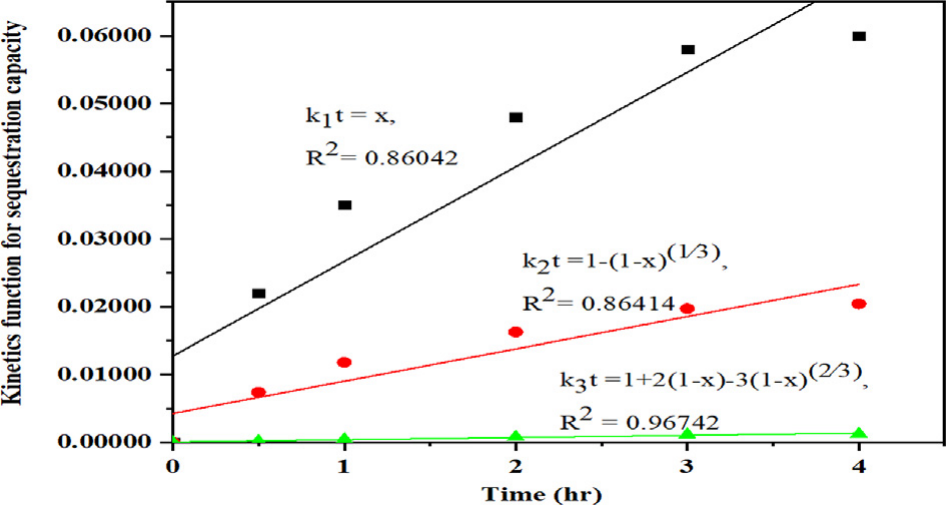
Carbon sequestration capacity of EAF steel slag against reaction time using the three types of kinetic equations.

The sequestration experimental data were analysed using Eqs. (8), (9), and (10) of the kinetic models by nonlinear regression. The calculated values were plotted, to find the best fitting model for the carbonation. It was found by calculating the R^2^ value for all the fitted models as shown in Fig. 11. Based on these values, the aqueous mineral carbonation process for the EAF slag was best controlled by the product layer phase thus, the values of $k_{3}t=1+2\left(1-x\right)-3{\left(1-x\right)}^{2/3}$ rather than by the chemical reaction (reactant) controlled phase at the solid surface, This gives the linear relationship for the sequestration at the ambient temperature with the coefficient correlation of R^2^ to be 0.96742, which shows that the reaction and the calcium carbonate formation was controlled by the ash layer product phase process (Jo et al., 2017; Tu et al., 2015; Ajemba and Onukwuli, 2012) and not the carbonic acid that was formed during the reaction between the CO_2_, and water that would have influence the leaching of the Ca ion from the slag.

## 4 Conclusion

This study shows that the EAF slag from iron and steel industry at Klang, Malaysia with 20.91weight % of CaO in the fresh slag composition was used for CO_2_ sequestration. Moreover, the BET, XRD, FTIR, EDX/SEM patterns confirmed the suitability of the material for CO_2_ capturing and storing. The experiment was conducted with CO_2_ gas pressure of 5 bar, and 5:1 L/S ratio in 3 h, with the EAF slag size of <63 μm at ambient temperature. The maximum CO_2_ uptake capacity obtained was 5.836 wt % which gives a sequestration of 58.36 g CO_2_/kg of steel slag and the sequestration efficiency of 32.53 %. The shrinking core model was used to describe the kinetics of using EAF slag for the carbon sequestration, which was controlled by the product layer phase. For higher CO_2_ sequestration and pure CaCO_3_ an indirect route of mineral carbonation could be used for further work. The EAF slag demonstrated to be a potential and feasible material for excess CO_2_ sequestration at moderate process conditions, as compared to many other steel slags. Hence, instead of using the wastes for landfill, it can be used for CO_2_ sequestration.

## Declaration

### Author contribution statement

Sunday Ogakwu Omale: Conceived and designed the experiments; Performed the experiments; Analyzed and interpreted the data; Wrote the paper.

Thomas S. Y. Choong: Conceived and designed the experiments; Analyzed and interpreted the data; Contributed reagents, materials, analysis tools or data; Wrote the paper.

Luqman C. Abdullah: Conceived and designed the experiments.

Shamsul I. Siajam, Mun W. Yip: Contributed reagents, materials, analysis tools or data.

### Funding statement

This work was supported by Universiti Putra Malaysia (Geran Putra IPS, GP-IPS/2017/9546100).

### Competing interest statement

The authors declare no conflict of interest.

### Additional information

No additional information is available for this paper.
